# Polymer–Inorganic Thermoelectric Nanomaterials: Electrical Properties, Interfacial Chemistry Engineering, and Devices

**DOI:** 10.3389/fchem.2021.677821

**Published:** 2021-04-26

**Authors:** Xiaoyan Zhang, Shuang Pan, Huanhuan Song, Wengai Guo, Shiqiang Zhao, Guang Chen, Qingcheng Zhang, Huile Jin, Lijie Zhang, Yihuang Chen, Shun Wang

**Affiliations:** College of Chemistry and Materials Engineering, Institute of New Materials and Industrial Technologies, Key Laboratory of Carbon Materials of Zhejiang Province, Wenzhou University, Wenzhou, China

**Keywords:** polymer-inorganic hybrids, nanomaterials, electrical properties, interfacial chemistry, thermoelectric devices

## Abstract

Though solar cells are one of the promising technologies to address the energy crisis, this technology is still far from commercialization. Thermoelectric materials offer a novel opportunity to convert energy between thermal and electrical aspects, which show the feasibility to improve the performance of solar cells *via* heat management and light harvesting. Polymer–inorganic thermoelectric nanocomposites consisting of inorganic nanomaterials and functional organic polymers represent one kind of advanced hybrid nanomaterials with tunable optical and electrical characteristics and fascinating interfacial and surface chemistry. During the past decades, they have attracted extensive research interest due to their diverse composition, easy synthesis, and large surface area. Such advanced nanomaterials not only inherit low thermal conductivity from polymers and high Seebeck coefficient, and high electrical conductivity from inorganic materials, but also benefit from the additional interface between each component. In this review, we provide an overview of interfacial chemistry engineering and electrical feature of various polymer–inorganic thermoelectric hybrid nanomaterials, including synthetic methods, properties, and applications in thermoelectric devices. In addition, the prospect and challenges of polymer–inorganic nanocomposites are discussed in the field of thermoelectric energy.

## Introduction

Thermoelectric materials represent a functional material capable of direct mutual conversion between heat and electricity. As an alternative strategy for conventional power generation, thermoelectric devices with less size, noise, and pollution have important application prospects (Wang et al., 2010). For instance, thermoelectric devices enable the effective utilization of the previously wasted heat, thus providing promising solutions for optimizing power generation technologies and improving fuel energy efficiency (Sales, 2002; Bell, 2008). Moreover, advanced techniques such as solar cells are also benefited due to the heat management and light harvesting of thermoelectric devices (Jurado et al., 2019; Xu et al., 2019). The corresponding thermoelectric performance is defined as thermoelectric figure of merit *ZT*: *ZT* = *S*^2^*T*σ/κ, where *S* is thermoelectric power or Seebeck coefficient, *T* is absolute temperature, σ is electrical conductivity, and κ is thermal conductivity. In order to obtain a large value of *ZT*, the material should possess high *S*, high σ and low κ. However, it remains a grand challenge to achieve all these features in one material as properties are interrelated. For example, based on the Wiedemann–Franz law, an increase in σ usually leads to larger κ (Carrete et al., 2012). To this end, hybrid thermoelectric materials such as polymer–inorganic nanomaterials have shown the feasibility to solve the issues of a single thermoelectric material.

Polymer–inorganic nanomaterials are an emerging and functional hybrid. They not only inherit the advantageous characteristics from each component such as high σ and *S* of inorganic materials, and low κ of polymeric materials, but they also possess novel interfacial chemistry. Their morphology, electrical features, and interfacial and surface chemistry play a vital role in the related application, which can be rationally designed *via* the well-established synthetic approaches. Due to these outstanding characteristics, polymer–inorganic nanomaterials can promote the value of *ZT* with enhanced compatibility, which leads to superior activity and stability in thermoelectric devices. In addition, *via* heat management and light harvesting, polymer–inorganic thermoelectric nanomaterials can also improve the efficiency of solar cells (Jurado et al., 2019).

In this review, we will highlight the development of polymer–inorganic thermoelectric nanomaterials, including the synthetic strategies, structures, electrical characteristics (thermoelectric properties), interfacial and surface chemistry, and applications in (solar) thermoelectric devices such as thermoelectric generators (TEGs), thermoelectric coolers (TECs), and thermoelectric sensors. The challenges of polymer–inorganic nanomaterials in thermoelectric devices will be discussed in depth.

## Interfacial and Surface Chemistry of Polymer–Inorganic Nanomaterials

Surface and interfacial chemistry is of fundamental importance in functional polymer–inorganic nanomaterials, which can be rationally designed and modified during synthetic stage (Marchetti et al., 2017). Benefiting from the development of synthetic strategies and the emerging technologies, polymer–inorganic nanomaterials with controllable dimension, composition and architecture have been designed and prepared. There are mainly four synthetic strategies: (1) assembly of polymer and inorganic material, (2) assembly of polymer and inorganic precursors, (3) assembly of organic precursor and inorganic material, and (4) assembly of organic and inorganic precursor.

These four strategies offer convenient tunability over interfacial and surface chemistry and thus properties. For example, for the polymer–inorganic nanomaterials prepared by the assembly strategy of polymer and inorganic material, the interface between each component is connected *via* relatively weak interfacial force. In contrast, the organic inorganic interface of the polymer–inorganic nanomaterials prepared by the polymer and inorganic precursor assembly strategy is well-defined, leading to better interfacial and surface chemistry and electrical characteristics in the following application.

## Electrical Properties of Polymer–Inorganic Thermoelectric Nanomaterials

The unique electrical properties of thermoelectric materials lie in their thermoelectric feature, which offers a novel opportunity to convert energy between thermal and electrical aspects. Their thermoelectric performance can be expressed *via* evaluating the figure of merit (*ZT*) (Majumdar, 2004; Jin et al., 2019). Although copious impressive work was devoted to developing thermoelectric materials, the *ZT* values are usually <1, still lower than the targeted values of 3 or higher. This challenge shows the difficulties to realize high σ, high *S*, and low κ in one material for high *ZT*. Inorganic nanomaterials usually exhibit high S and σ (Zhang et al., 2020b). However, increased σ is usually associated with enhanced κ in conventional inorganic materials based on the Wiedemann–Franz law as the density, migration, and scattering of charge carriers are interrelated with heat transfer and thermal energy (Carrete et al., 2012). Meanwhile, inorganic reagents are relatively expensive. On the other hand, polymeric thermoelectric materials have low σ, *S*, and power factor (*S*^2^σ). Therefore, thermoelectric materials consisting of polymer exhibit *ZT* values several orders of magnitude lower than those of inorganic ones. However, polymeric materials possess peculiar features like low density, cost and κ, and capability of large-scale preparation (Zhang et al., 2014). Clearly, it is highly desirable to design and craft polymer–inorganic nanohybrids integrating high σ and *S* of inorganic materials, and low κ of polymeric materials.

**Figure 1:**
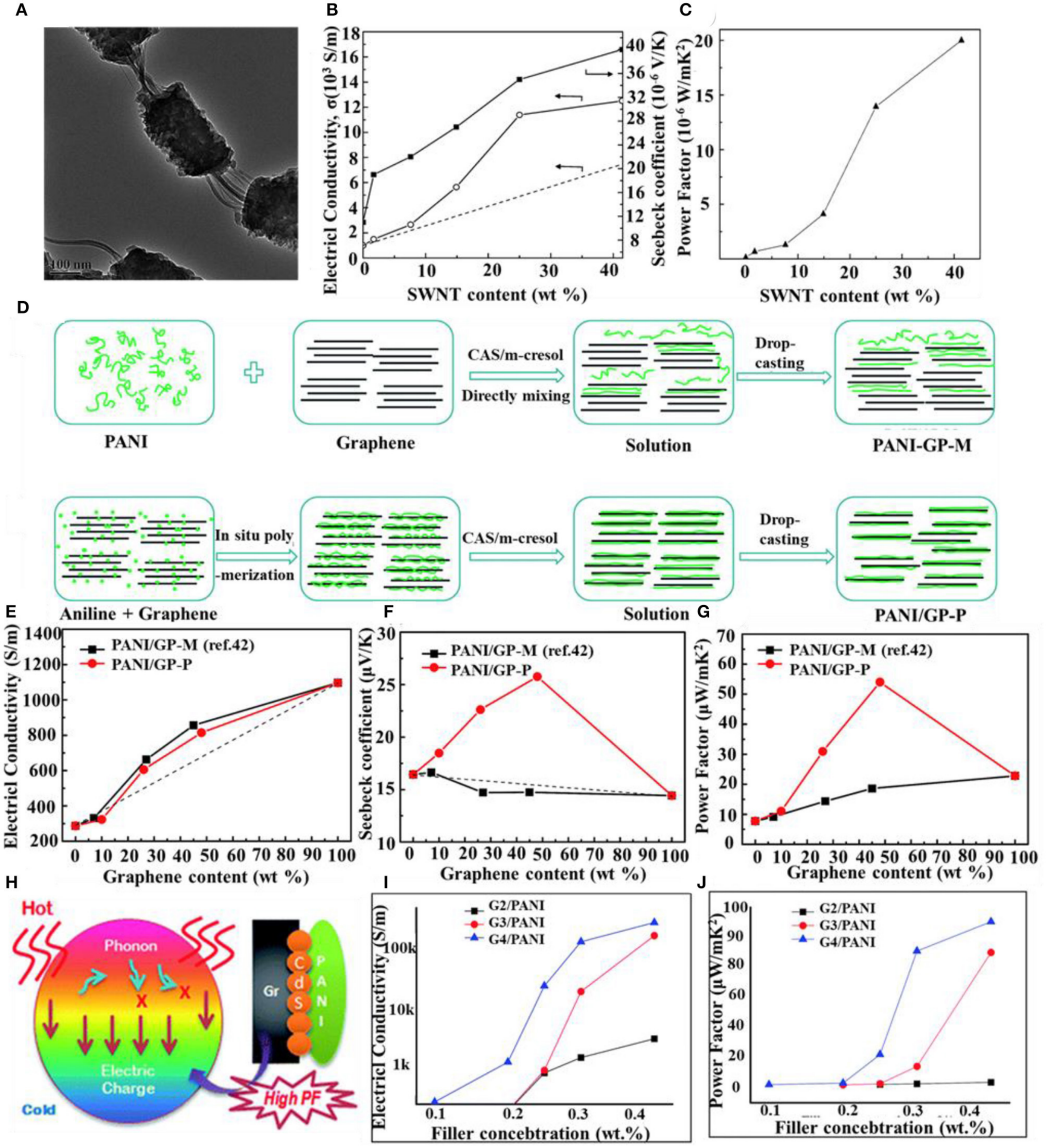
**(A)** TEM image for SWNT/PANI composites containing 25 wt% SWNT, **(B)** Seebeck coefficient (closed squares), electrical conductivity (open circles), and **(C)** power factor (closed triangles) of SWNT/PANI composites containing different SWNT content. The dashed line is the calculated electrical conductivity based on the particle mixture rule. **(D)** Schematic illustrations of the synthesis procedure of (top) PANI/GP-M and (bottom) PANI/GP-P composite films and their corresponding **(E)** in-plane electrical conductivity, **(F)** Seebeck coefficient, and **(G)** power factor at room temperature with different graphene contents. **(H)** Schematic representation of RGO/CdS/PANI preparation nanocomposites for thermoelectric applications, **(I)** Seebeck coefficient, and **(J)** power factor vs. filler concentration. Reproduced with permission.

Polyaniline is a typical conducting polymer with a conductivity of up to 10^5^ S m^−1^ and good stability. However, its power factor is low. Recently, researchers have added inorganic nanomaterials to improve its power factor. Up to now, there have been several reports on polyaniline-inorganic thermoelectric composite materials. For example, Yao et al. (2010) synthesized *in situ* single wall carbon nanotubes/polyaniline (SWNT/PANI) polymer–inorganic nanohybrids using single wall carbon nanotubes (SWNT) as templates (Figure 1A), which directed the polyaniline to grow along the surface of CNT forming an ordered chain structure. Compared with pure PANI, SWNT/PANI nanohybrids displayed higher electrical conductivity and Seebeck coefficient up to 1.25 × 104 S m^−1^ and 40 μV K^−1^ (Figure 1B), respectively, with maximum power factors being as high as 2 × 10^−5^ W m^−1^K^−2^ (Figure 1C). This performance improvement could be ascribed to the interfacial and surface chemistry (strong π-π interactions) of SWNT/PANI.

Graphene is also one type of preferred nanofillers due to its large surface area and aspect ratio (Hasani et al., 2020). Compared with SWNT, graphene is easier to prepare at lower cost, which facilitates the practicability of the final thermoelectric product (Liu et al., 2019). To this end, Wang et al. (2015) prepared polyaniline/graphene (PANI/GP) polymer–inorganic nanohybrids by *in situ* polymerization and solution method (Figure 1D). During *in situ* polymerization, polyaniline covered the graphene surface with intense π-π conjugation interactions, resulting in the formation of improved molecular ordering in the nanohybrid. Therefore, the thermoelectric properties of the composite were significantly enhanced with an optimal electrical conductivity of 814 S cm^−1^ (Figure 1E) and a Seebeck coefficient of 26 μV K^−1^ (Figure 1F), and the maximum power factor was 55 μW m^−1^ K^−2^ (Figure 1G). Graphene oxide is another effective material to improve the thermoelectric properties of polyaniline. After hybridization, the electrical conductivity, Seebeck coefficient, and power factor reached around 1,489 S cm^−1^, 59 μV K^−1^, and 52.11 × 10^−7^ W K^−2^ cm, with a maximum *ZT* of 0.8 at 363 K (Shalini et al., 2019).

In addition to carbon materials, metal-based inorganic materials have a large Seebeck coefficient, such as Bi_2_Te_3_ (Li et al., 2011), Sn_0.85_Sb_0.15_O_2_ (Plochmann et al., 2014), PbTe (Wang et al., 2010), Ag_2_Te (Wang et al., 2012), Cu_2_Te (Zhou et al., 2015), PtTe (Zhang et al., 2020), and Au (Toshima et al., 2012). Therefore, these inorganic materials can be combined with conducting polymers to improve the thermoelectric properties. For example, More et al. (2017). prepared RGO–CdS–polyaniline (PANI) ternary hybrid polymer–inorganic nanohybrids by a simple *in situ* thermoelectric method. As shown in Figures 1H–J, the energy band arrangement between reduced graphene oxide (RGO), CdS quantum dots (QDs), and PANI improved the P-type electrical conductivity with reduced thermal conductivity and proved the synergetic energy filtering effect. The electrical conductivity and power factor of RGO/CdS/PANI nanohybrids increased with the increase of filler concentration (RGO–CdS). The nanohybrid (with 0.4 wt% filler; G4) had low thermal conductivity and high electrical conductivity, and its *ZT* value was as high as 1.97.

## Applications in Thermoelectric Devices

Thermoelectric materials are usually assembled into thermoelectric devices for use in the energy field, which efficiently takes advantage of the low-quality heat that otherwise is dissipated. Thus, thermoelectric devices have received increasing attention from the viewpoints of both scientific research and technological development. The past several decades have witnessed a rapid advance of high-performance thermoelectric devices including TEGs, TECs, and thermoelectric sensors, which will be highlighted in the following sections.

### Thermoelectric Generators

A TEG is a kind of device that directly generates electrical energy from heat. Generally (Sales, 2002; Bell, 2008), various thermal energy sources such as natural heat (e.g., solar radiation) and waste heat (e.g., industrial production), which induces a temperature gradient and can be capitalized on for energy generation in a reliable, pollution-free, and eco-friendly way (Mulla et al., 2020). Since many low-quality heat sources display irregular shapes and surfaces, it is vital for TEGs to be flexible for tight contact with the heat sources and thus maximizing heat collection. Such flexible TEGs have also gained increasing interest for advancing the development of many self-powered devices [e.g., smartwatches and smart and flexible wearable electrocardiograms (Dargusch et al., 2020)].

**Figure 2:**
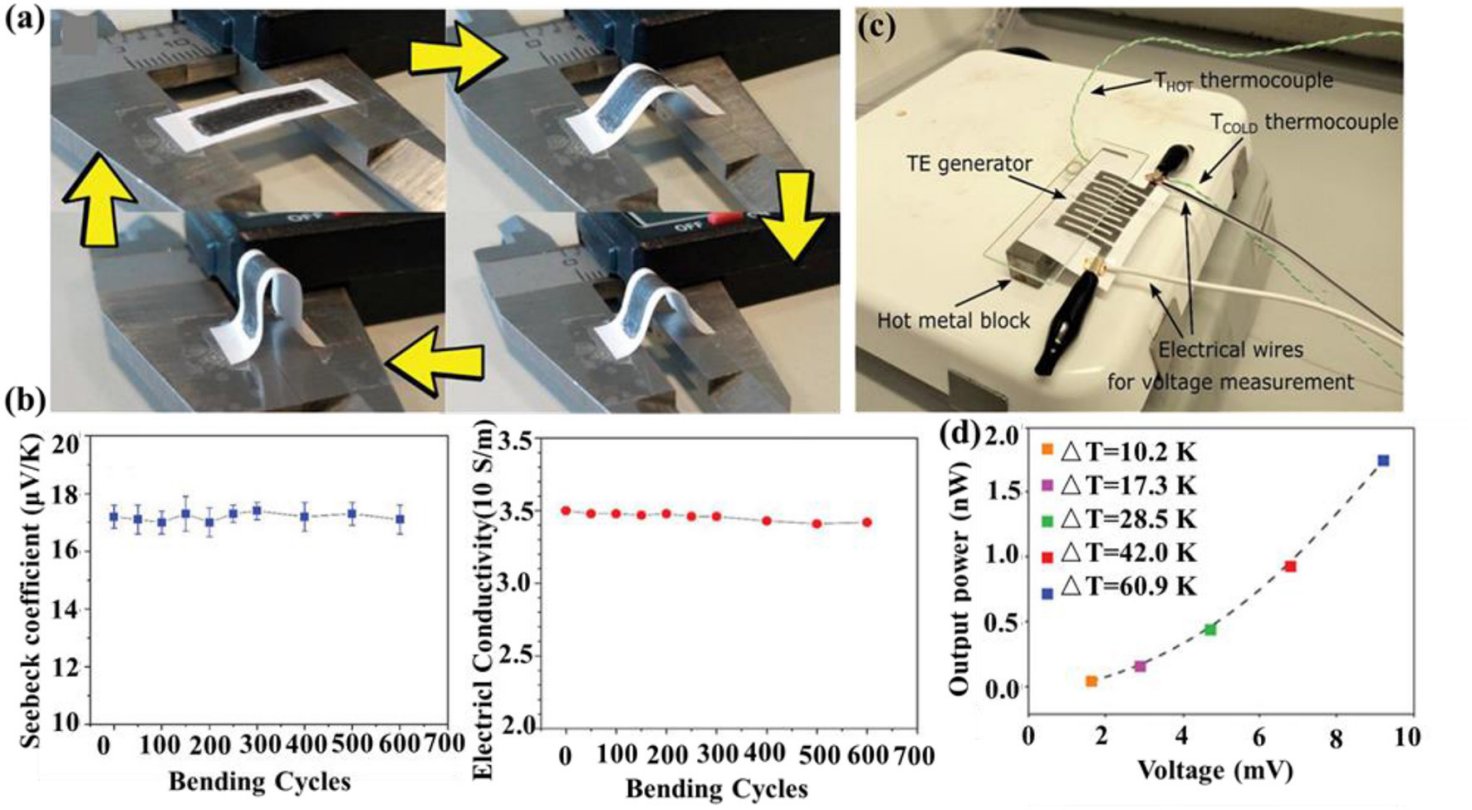
**(a)** Photographs of the cyclical bending procedure applied to the thermoelectric trace with individual p-type and n-type graphite traces and **(b)** Seebeck coefficient (left) and electrical conductivity (right) over multiple bending cycles. **(c)** Photograph of the setup used for electrical testing of the thermoelectric generator (TEG) module using graphite and polyethylenimine and **(d)** the corresponding power output vs. voltage with respect to ΔT. Reproduced with permission.

To minimize the cost of energy cost with optimal performance, polymer/inorganic nanohybrids with additional interfacial and surface chemistry have been developed as promising thermoelectric materials. For example, Mulla et al. (2020) successfully fabricated flexible paper-based TEGs using graphite and polyethylenimine (Figure 2). Graphite served as both p-type and n-type thermoelectric legs, which did not require additional paste for connection. The thermoelectric voltage and corresponding output power reached 9.2 mV and 1.75 nW, respectively, at a temperature difference of about 60 K. Flexible TEGs with optical transparency exert a crucial impact on novel applications such as smart windows and solar cells (Chowdhury et al., 2009). The key lies in the development of transparent p-type thermoelectric materials. To this end, Yang et al. deposited transparent p-type γ-Cui onto the PET substrate for single-leg thermoelectric device. The corresponding maximum output power at ΔT of 10.8 K was 8.2 nW with a power density of 0.1 mW cm^−2^, comparable with those of devices based on Bi_2_Te_3_/Sb_2_Te_3_ (Yang et al., 2017).

Rational design in architecture can further improve the thermoelectric performance of flexible TEGs. For example, the thermal energy loss generated by commercial substrate (e.g., Si, paper, and polymer film) significantly restricts the energy generation efficiency. To this end, Kim et al. (2014) developed a wearable TEG on a glass fabric without top and bottom substrates. The flexible device possessed a thin thickness of ~500 μm and a lighter density of ~.13 g cm^−2^ enabled by screen printing technology. Due to the unique design, the TEG displayed a high output power density, which is one order of magnitude higher than that of the previously reported flexible TEG.

Conducting polymers [e.g., poly(3,4-ethylenedioxythiophene)/poly(styrenesulfonate) (PEDOT:PSS) and poly(3-hexylthiophene) (P3HT)] with high flexibility and low thermal conductivity are ideal materials for fabricating flexible TEGs (Chabinyc, 2014). Song and Cai (2017) *in situ* synthesized PEDOT:PSS functionalized Te nanorod [phenol–formaldehyde–Te (PF–Te)], which were incorporated with PEDOT:PSS forming PEDOT:PSS/PF–Te composite film via vacuum-assisted filtration method. Through the thermoelectric performance test, it was found that the conductivity of the composite material increased to 1,262 S cm^−1^, and the power factor reached 51.4 μW m^−1^K^−2^. PEDOT:PSS/PF-Te composite film was then used to fabricate a composite prototype generator consisting of eight single-leg modules pasted onto a polyimide substrate. At a temperature difference of 13.4 K, an output voltage of 2.5 mV was achieved. These investigations have shown promising feasibility for tremendous applications such as wearable devices and smart systems *via* advancing the development of new thermoelectric materials and novel TEG designs.

The relatively small temperature difference from body heat hinders its practical application (Jung et al., 2017). Therefore, TEG with solar energy as a heat source (i.e., solar TEGs and STEGs) has attracted increasing interest. In this regard, the conversion of sunlight to electricity is mainly achieved by photovoltaic and solar thermal power generation (Kraemer et al., 2011; Wang et al., 2019). The solar cell represents a promising technique to solve energy crisis (Jiang et al., 2020; Zhang et al., 2020a). Thermoelectric materials in STEGs have two advantages: (a) TEGs can act as a cooling system to decrease the temperature, which improves the performance of solar cells (Benghanem et al., 2016). (2) TEGs utilize the dissipated heat energy for additional electricity generation (Kil et al., 2017). Based on this, Jurado et al. (2019) studied STEGs using polymer–CNT composites as both solar absorbent and active TE materials. Due to the effect of interfacial chemistry and solar thermoelectric synergy effect, STEG with a power output of 180 nW was obtained.

### Thermoelectric Coolers

The basic principle of TECs follows the Peltier effect to generate endothermic or exothermic phenomena between the interfaces of two different materials. During the past few decades, TEC has garnered increasing attention in academia and industries due to its simple construction, reliability, flexibility, noiseless operation, and long life. Localized cooling enabled by wearable TECs can gradually take the place of conventional systems, which decreases climate warming and power cost (Kishore et al., 2019). Hu et al. (2020) prepared polymer–inorganic nanohybrid film consisting of nickel nanowires evenly dispersed in PVDF matrix by solution mixing casting and compression molding. The electrical conductivity of the film raised as a function of Ni NW content while the optimal power factor reached 24.3 μWm^−1^K^−2^. The flexible thermoelectric thin film has enormous potential in the TECS. Sun et al. (2012) fabricated a thermoelectric module containing 35 n-type couples of poly[Na_x_(Ni-ett)] and p-type poly[Cu_x_(Cu-ett)] (ett: 1,1,2,2-ethenetetrathiolate), which generated output voltage, current, and power of 0.26 V, 10.1 mA, and 2.8 μW cm^−2^, respectively, with good stability at a temperature difference of 80 K. The single thermocouple exhibited cooling effect with a ΔT of 3.5 K at an external voltage of 0.6 V.

Batteries usually generate a lot of heat during rapid charging and discharging cycles, which causes high temperatures and even battery explosions (Xie et al., 2020). Therefore, temperature control is important for safe battery operation (Kai et al., 2018). In a water-based electrolyte, the water molecules can form extrathermodynamic cycle via evaporation and solidification to realize the heat absorption/release cycle during thermoelectric conversion (Zhang et al., 2017). With this in mind, Chen et al. designed an intelligent hydrogel film consisting of polyacrylamide (PAAm) and ${\text{Fe}(\text{CN})}_{6}^{3-}$ and ${\text{Fe}(\text{CN})}_{6}^{4-}$. The results showed that TG hydrogel reduced battery temperature by 6.5, 13.2, 15.7, and 20°C at discharge rates of 1.6, 1.8, 2, and 2.2°C, respectively (Pu et al., 2020).

### Thermoelectric Sensors

In addition to TEGs and TECs, the thermoelectric properties have also prompted the development of thermoelectric sensors, which release combustion heat during chemical reaction with gas. Currently, thermoelectric sensors based on a catalyst combustor have realized the monitoring of various chemical gases with a wide range (e.g., 0.5 ppm to 5 vol% for H_2_, 1 ppm for CH_4_) (Nishibori et al., 2009; Nagai et al., 2015). However, even though these devices are efficient, their cost and weight are relatively high. To address these, polymer–inorganic thermoelectric nanohybrids served as alternative thermoelectric sensors. Slobodian et al. (2016) designed one self-powered thermoelectric sensor consisting of oxidized multi-walled carbon nanotubes and ethylene-octene copolymer (MWCNT/EOC). The thermoelectric power became larger as a function of the generated oxygen-containing functional groups on the MWCNT surface. The resistance of MWCNT/EOC composite material was affected by organic vapor, with average increases of 3.6, 1.1, and 0.05 S upon exposure to heptane, toluene, and ethanol saturated vapor, respectively. Such a device has provided a platform for convenient gas alarm and detection.

## Summary and Perspectives

In summary, the recent advancement of polymer–inorganic thermoelectric nanomaterials is highlighted. After an introduction of synthetic strategies, interfacial chemistry engineering and electrical properties are presented. The development of thermoelectric devices, namely, TEGs, TECs, and thermoelectric sensors is also summarized. In particular, the burgeoning integration of solar cells with the thermoelectric technique is discussed.

However, despite the copious advances achieved by polymer–inorganic thermoelectric nanomaterials, in-depth explorations are still necessary due to the remaining challenges. For example, the substantial distinctions between inorganic and organic materials regarding intrinsic nature may cause phase and charge separation induced within the interface. In addition, due to the restricted transportation of charge carriers over the interface between phases with dissimilar electrical properties, a local polarization may generate an electric field much larger than the applied one. Thus, the polymer–inorganic thermoelectric nanomaterials may suffer from quicker destruction. To circumvent these issues, delicately balancing the intrinsic distinct properties between organic and inorganic components is needed for an optimized characteristic feature and practical behavior of polymer–inorganic nanohybrids. The following aspects for the development of polymer–inorganic thermoelectric nanomaterials might deserve more attention: (1) Adjusting the architectures and size of inorganic components may lead to additional quantum effects, which is conducive to the charge transport process. (2) Development of synthetic approaches that are capable of generating polymer–inorganic thermoelectric nanomaterials with well-defined interfacial and surface chemistry is of great importance, such as the polymer-enabled nanoreactor strategy (Li et al., 2018). (3) Surface of each component is better to be functionalized with specific ligands to improve the overall compatibility, therefore enhancing the stability and electric properties of nanohybrids. (4) Experimental results are expected to combine with powerful *in situ* characterizations and theoretical modeling (Yu et al., 2020) which facilitates the comprehensive insight of structure–property–performance relationship.

With the significant progress and tremendous effort in this emerging area, we envision that many breakthroughs of polymer–inorganic thermoelectric nanomaterials will promote their use in the near future.

## Author Contributions

All authors listed have made a substantial, direct and intellectual contribution to the work, and approved it for publication.

## Conflict of Interest

The authors declare that the research was conducted in the absence of any commercial or financial relationships that could be construed as a potential conflict of interest. The reviewer XL declared a past co-authorship with one of the authors YC to the handling Editor.
